# Exit Antonius

**Published:** 1903-05

**Authors:** 


A Monthly Review of Medicine and Surgery.

*EDITOR:*

WILLIAM WARREN POTTER, M. D.

All communications, whether of a literary or business nature, books for review and exchanges should be addressed to the editor: 284 FRANKLIN STREET, BUFFALO, N. Y.

VOL. XLII.—LVIII. MAY, 1903. No. 10.

### Exit Antonius.

THE most abandoned medical fakir that Buffalo has ever tolerated has been tried, found guilty and sentenced to imprisonment for conspiracy to defraud. Nearly two years ago a person calling himself "Antonius, the Boy Wonder," took up his residence in this city, and opened an "Institute of Science," where he carried on the business of pretending to heal the sick. In this enterprise he employed a physician legally authorised to practise medicine, presumably to protect himself against arrest. He gave public exhibitions of his pretense at a public hall, and boldly advertised his brazen scheme of deceit and fraud in the daily newspapers. And the newspapers published his statements that on their face were false!

There were, however, two notable exceptions, for the *Buffalo Review* and the *Catholic Union and Times* declined to enter the literary bargain of deception. It is to that sterling newspaper, *The Buffalo Review*, that the people are indebted for the conviction of this audacious villain. Under a former administration of the district attorney's office attempts had been made to bring Antonius to trial, but in vain. With the election of Mr. Edward E. Coatsworth to the office of district attorney last November, a new régime was instituted in that office. The *Review* at once began a crusade against the prince of fakirs, which was conducted daily in the columns of that paper, unfolding his schemes and branding his advertisements as falsehoods.

In February the grand jury returned three indictments against Anton J. Weichers, *alias* Antonius, the Boy Wonder (aged 39 years), and he was brought to trial in the March term of the criminal court, Mr. Justice Kruse, presiding. The indictment selected was for conspiracy to defraud, and the prosecution was ably conducted by Mr. Frank A. Abbott, first assistant district attorney. The trial lasted five days and the culprit was defended by Mr. E. M. Bartlett in a masterful manner, every known expedient being resorted to to stay the hand of justice; but in vain! The jury appeared to have no difficulty in coming to a decision, the evidence being overwhelming, and a verdict of guilty quickly followed the able charge of the judge.

In imposing sentence Justice Kruse said so much that is of importance and general interest and, withal, his analysis of the case as set forth in the sentence is such an indictment against all who aided and abetted the fakir, that we print it in full as follows:

By whatever name you may have seen fit to have called the practice in which you were engaged, I am quite satisfied that it was all permeated with fraud and deception. There may have been isolate cases where people have received some relief from the physicians that you have had with you, but I think in general you have taken money from people, to which you were not entitled.

The practice adopted by you was well calculated to deceive the credulous and simple-minded. Many of them were of that class of people who are easily imposed upon, and if there is any class of people above another that the law should shield and protect from imposters, like yourself, it is that class of people. As they came upon the stand, one after another, some of whom testified for as well as against you, their condition excited pity. Their simplicity was pathetic. To their burden of chronic ailments in many instances was added that of poverty. You took their money and, in my judgment, you were not entitled to it.

It is, however, true that you have simply been convicted of a conspiracy to commit a fraud in connection with others, and not of the fraud itself, which of course is not so serious a crime as where a person actually commits the fraud. While the evidence does show that you did actually take money, yet that was not the crime for which you were being tried, although it was brought out in the testmony tending to show the unlawful scheme and conspiracy which you and your confederates hit upon, for the purpose of getting this money.

In the view which I take of your conduct, therefore, I can't be lenient in this case. The suggestion has been made by some of your friends that a fine ought to meet the requirements. I can't take that view of it. The law does not permit people like yourself to impose upon persons whose horizon in life has been narrow and circumscribed, and who are most easily imposed upon. It is necessary, in the interests of justice, that I should impose imprisonment and the sentence of the court is that you be confined in the Erie County Penitentiary for the period of nine months.

Dr. Franklin S. Temple, who was lately an assistant to or co-worker with Antonius, was arrested about the middle of February on a bench warrant, based on three indictments found by the grand jury. One indictment charged him with grand larceny, another with conspiracy, and the third with practising medicine under an assumed name. His bail was fixed at $2,500, in default of which he was sent to jail, where he remained until he and his co-conspirator Antonius, the boy Wonder (aged 39 years), were arraigned for trial. Temple pleaded guilty, and after the trial and sentence of Antonius, Judge Kruse suspended sentence upon Temple. The remaining medical fakirs in Buffalo should take heed from these two cases. If they do not cease their fraudulent practices, there are a few vacant rooms in the penitentiary awaiting them on Front Avenue.

H. H. Bridgewater, who attempted to carry on "business" for Antonius, while he was undergoing trial, was fined $50 by Judge Murphy in Police Court, April 1, 1903, after being convicted of violating the health laws. The complainant in the case was the Medical Society of the County of Erie, which accused Bridgewater of illegally assuming the title of physician and practising medicine without a license. Being without funds, he was sent to the penitentiary for 50 days.

This is another notice served on the violators of the laws relating to the practice of medicine in this state. It would be well for them to understand at once that there will be a vigorous prosecution of each and all who transgress the laws of the state in spirit or in letter, so far as they relate to the practice of medicine.

